# Supplementary material for: Chromatin retained MUSHER lncRNA integrates ABA and DOG1 signalling pathways to enhance Arabidopsis seeds dormancy
Source: Nat Commun. 2025 Aug 14;16:7545. doi: 10.1038/s41467-025-62991-5 (PMC12354759; doi:10.1038/s41467-025-62991-5)
Supplement: Supplementary file 1 — Supplementary Information [file 41467_2025_62991_MOESM1_ESM.pdf]

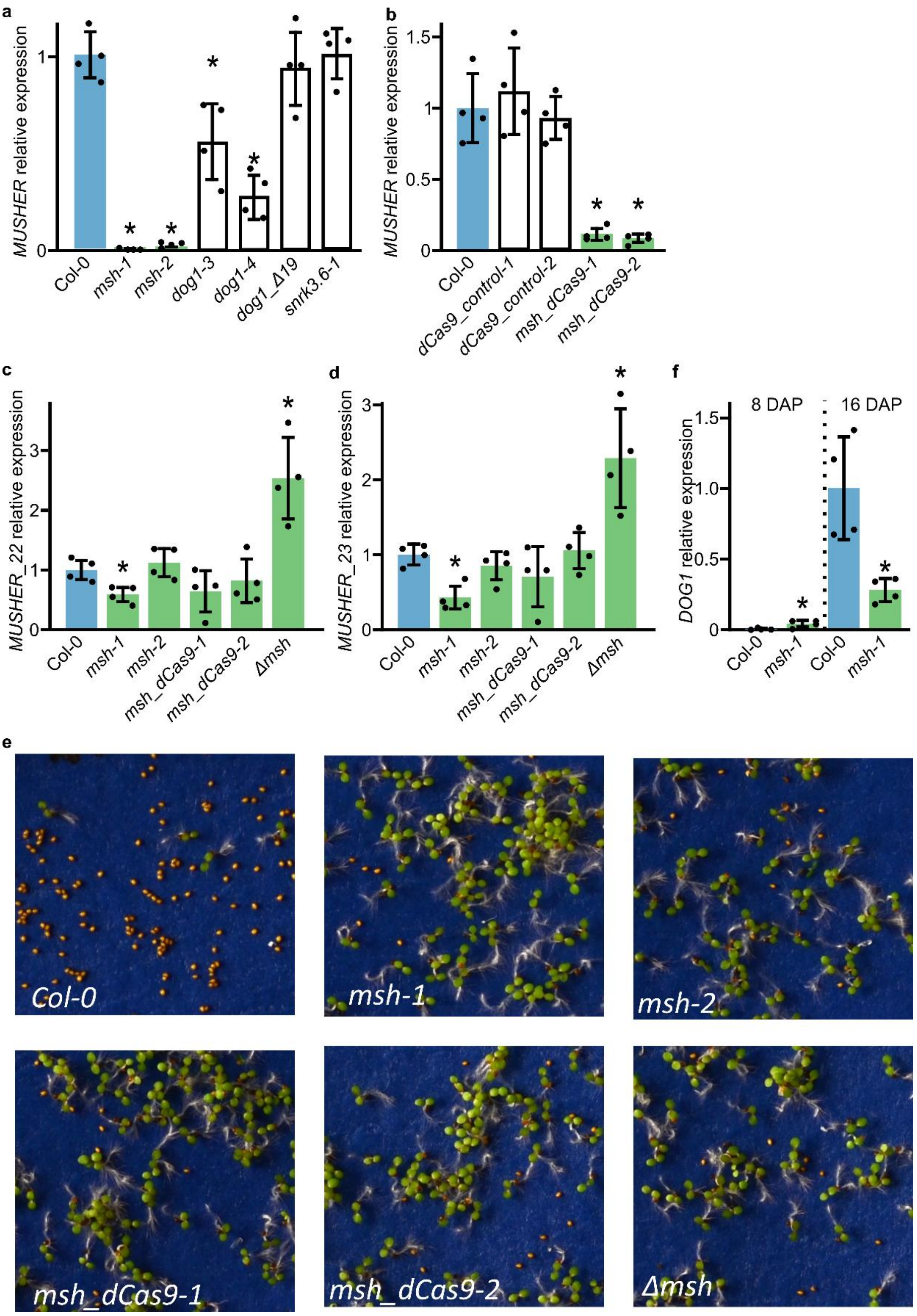
a
MUSHER relative expression
1
0
*
Col-0
msh-1
msh-2
dog1-3
dog1-4
dog1_Δ19
snrk3.6-1
b
MUSHER relative expression
1.5
1
0.5
0
Col-0
dCas9_control-1
dCas9_control-2
msh_dCas9-1
msh_dCas9-2
c
MUSHER_22 relative expression
3
2
1
0
Col-0
msh-1
msh-2
msh_dCas9-1
msh_dCas9-2
Δmsh
d
MUSHER_23 relative expression
3
2
1
0
Col-0
msh-1
msh-2
msh_dCas9-1
msh_dCas9-2
Δmsh
f
8 DAP
16 DAP
DOG1 relative expression
1.5
1.0
0.5
0
Col-0
msh-1
Col-0
msh-1
e
Col-0
msh-1
msh-2
msh_dCas9-1
msh_dCas9-2
Δmsh

1

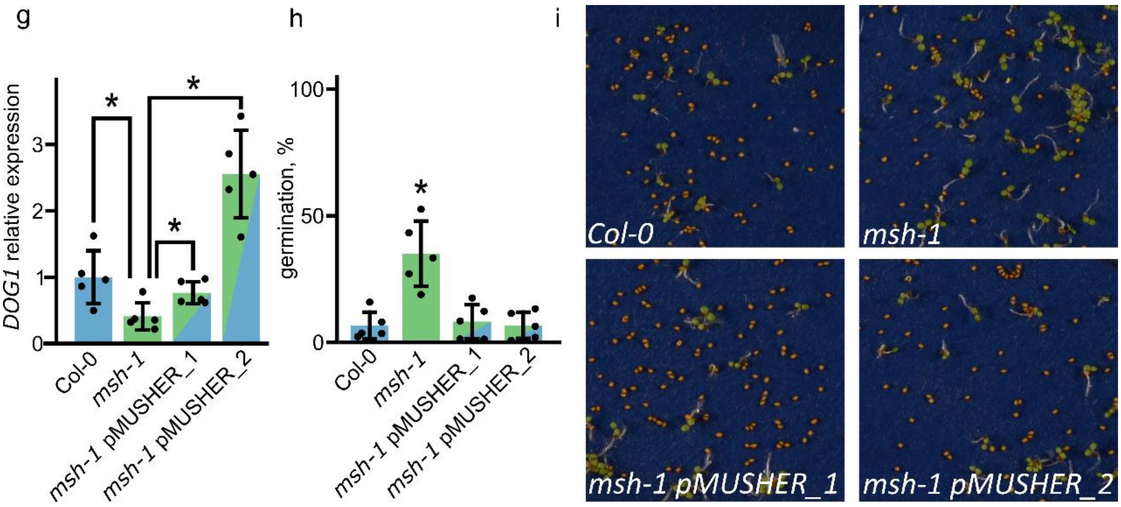


**Supplementary Fig. 1| *MUSHER* expression is partially dependent on *DOG1*.**

**a** *MUSHER* relative expression level in developing seeds of WT and indicated mutants, normalised to *UBC21* and relative to WT. *p*-value was calculated in comparison with WT. *p < 0.05. Data are presented as mean value of five biological replicates +/- SD. **b** *MUSHER* relative expression level in freshly harvested seeds of WT and dCas9 mutants, normalised to *UBC21* and relative to WT. *p*-value was calculated in comparison with WT. *p < 0.05. Data are presented as mean value of five biological replicates +/- SD. New transcripts around *MUSHER locus* were quantified using *MUSHER_2* **(c)** and *MUSHER_3* primers **(d)**. Transcript relative expression level in WT and indicated mutants seeds, normalised to *UBC21* and relative to WT. *p*-value was calculated in comparison with WT. *p < 0.05. Error bars represent the standard deviation of four biological replicates. **e** Representative photographs of *Col-0*, *msh-1*, *msh-2*, *msh_dCas9-1*, *msh_dCs9-2* and *Δmsh* corresponding to germination tests on Fig. 1f. **f** *DOG1* expression level during silique development in WT and *msh_dCas9-1* plants, normalised to *UBC21* and shown at the 8th and 16th days after pollination (DAP); Data are presented as mean value of three biological replicates +/- SD. **g** Relative expression of *DOG1* in freshly harvested seeds (primary dormancy) of WT, *msh-1* and *msh-1,* and *msh-1 MUSHER-complemented lines (msh-1 pMUSHER_1* and *msh-1 pMUSHER_2)*, normalised to *UBC21* and relative to WT. Data are presented as the mean value of five biological replicates +/- SD. **p*<0.05. **h** Freshly harvested seeds of WT, *msh-1* mutant and *msh-1 MUSHER-complemented lines (msh-1 pMUSHER_1* and *msh-1 pMUSHER_2)* were scored for germination. Germination was defined as radical protrusion and counted 4d after sowing. A two-tailed *t-test p*-value was calculated compared to WT (n=5, *p < 0.05). **i** Representative photographs of *Col-0*, *msh-1*, *msh-1 pMUSHER_1* and *msh-1 pMUSHER_2* corresponding to germination tests on Supplementary Fig. 1h.

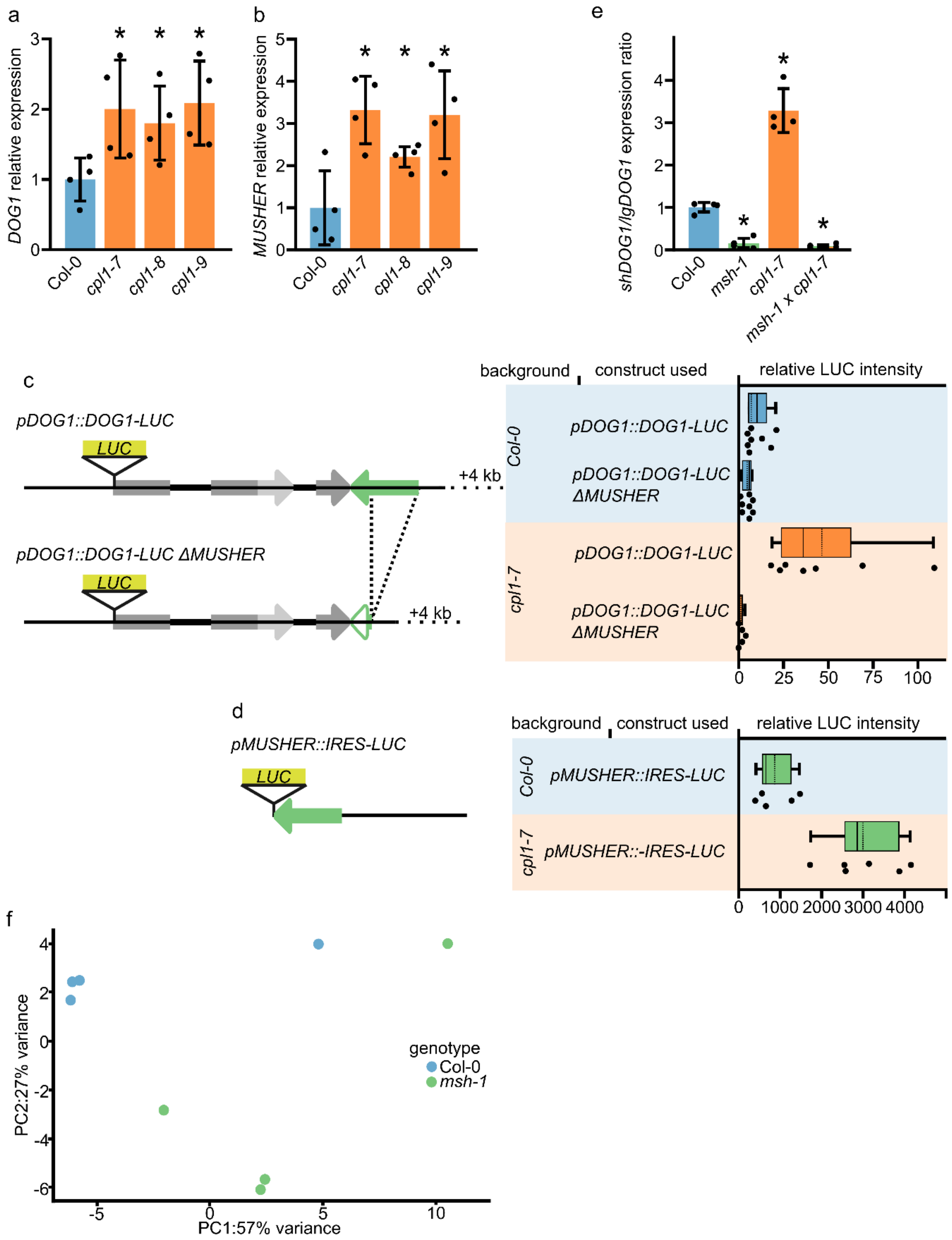


**Supplementary Fig. 2| *cpl1-7* mimics *MUSHER* overexpression leading to upregulation of *DOG1***

**a** *DOG1* and **b** *MUSHER* relative expression level in seeds of WT and *cpl1-7* mutant, normalised to *UBC21* and relative to WT. *p*-value was calculated in comparison with WT (**p*-value < 0.05, two-tailed t-test). Data are presented as the mean value of four biological replicates +/- SD. **c** Luciferase (LUC) gene reporter assay was used to measure *MUSHER's* ability to activate the *DOG1* gene expression. Schematic diagram showing regions included in *LUC* gene fusion constructs used for transient transformation of Arabidopsis WT and *cpl1-7* mutant seedlings. In boxplots, middle lines show the medians (solid line) and mean (dotted line); box limits indicate the 25th and 75th percentiles; whiskers extend 1.5 times the interquartile ranges. ($n_{cpl1\text{-}7 pDOG1::DOG1\text{-}LUC}$=7,for others n=8,* *p*-value < 0.05). **d** Luciferase activity assay on

transgenic seeds containing the *LUC* gene fused with the *MUSHER* promoter. In boxplots, middle lines show the median (solid line) and mean (dotted line); box limits indicate the 25th and 75th percentiles; whiskers extend 1.5 times the interquartile ranges. ($n_{Col\text{-}0}$=5, $n_{cpl1\text{-}7}$=6,* $p$-value < 0.01. **e** The ratio of *shDOG1* and *lgDOG1* isoforms in freshly harvested seeds of WT and *msh-1, cpl1-7* and double *msh-1 cpl1-7* mutants, normalised to *UBC21* and relative to WT (two-tailed t-test, *$p$-value < 0.05). Data are presented as the mean value of four biological replicates +/- SD. **f** PCA analysis of four biological replicates used in 3'RNA-seq analysis.

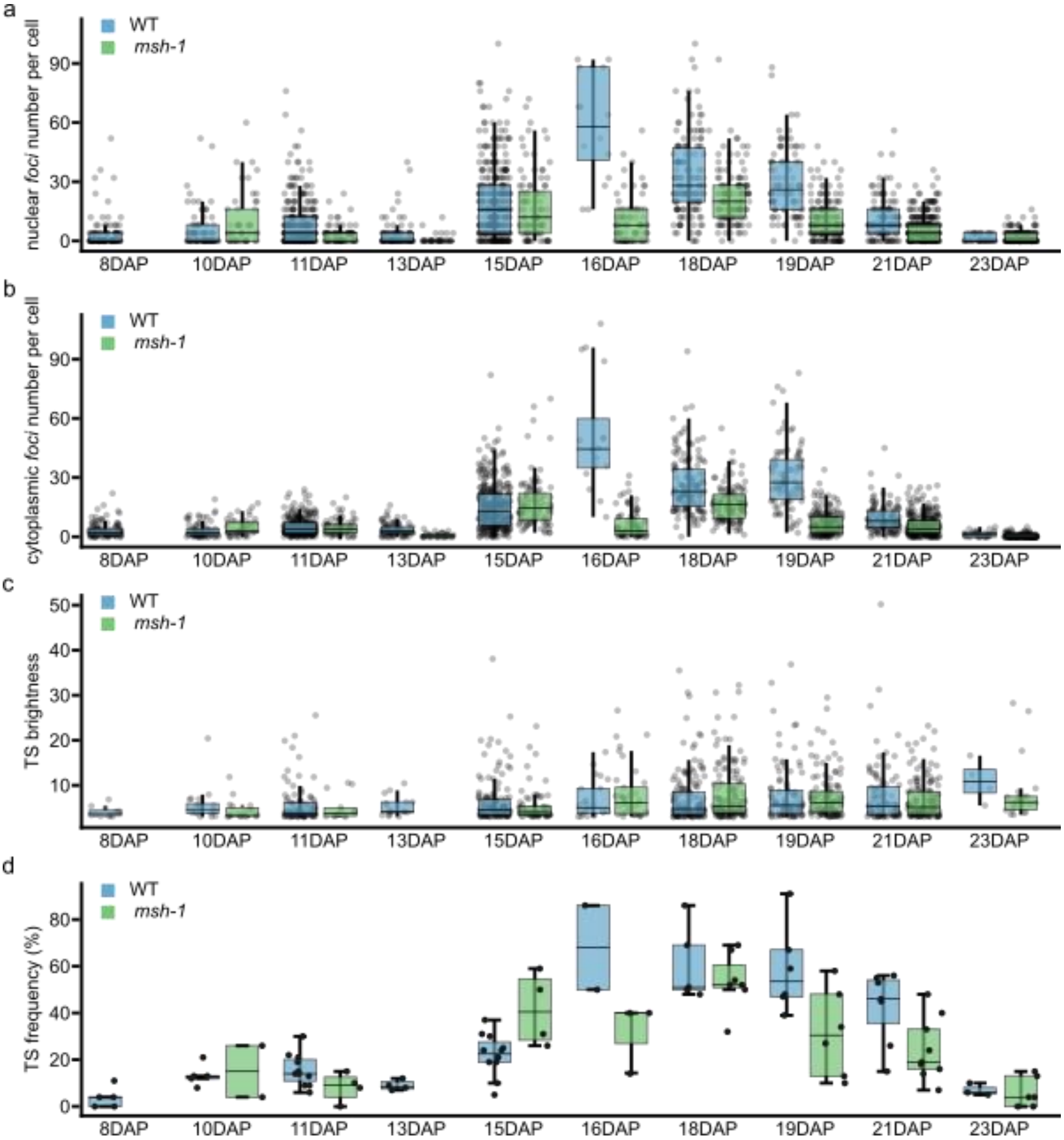


**Supplementary Fig. 3| *MUSHER* promotes *DOG1* mRNA abundance in the nucleus and cytoplasm but not burst frequency or intensity.**

**a** Nuclear *DOG1* mRNA foci number per cell during silique development in WT and *msh-1*. **b** Cytoplasmic *DOG1* mRNA foci number per cell during silique development in WT and *msh-1*. **c** Intensity of *foci* corresponding to *DOG1* transcription sites (TS) during silique development in WT and *msh-1*. *Foci* with a fold-change ≥3 times the average intensity of all nuclear foci, were considered as TS. **d** Frequency of *DOG1* TS during silique development in WT and *msh-1*. For all box plots, the middle line represents the

median; the upper and lower lines are the first and third quartiles (Q1 and Q3); whiskers extend 1.5 times the interquartile ranges. The x-axis represents days after pollination (DAP).

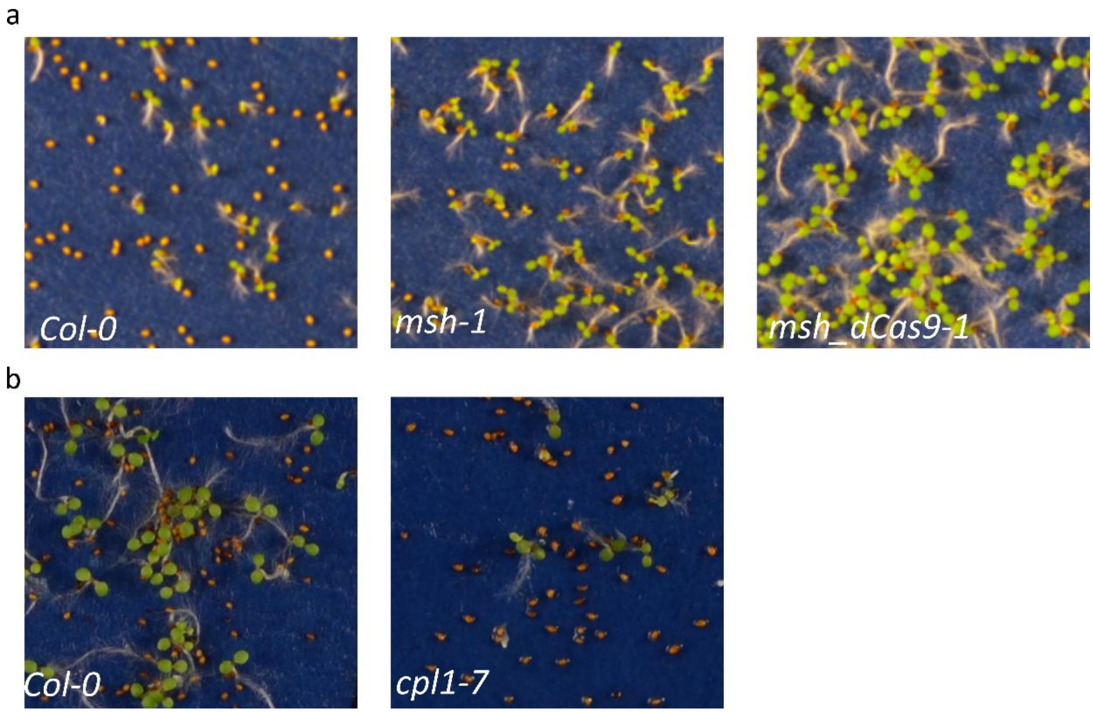


**Supplementary Fig. 4| Lower secondary dormancy of *MUSHER* mutants and higher secondary dormancy in *cpl1-7*.** **a** Representative photographs of WT, *msh-1* and *msh_dCas9-1* seeds corresponding to germination tests on Fig. 3a. **b** Representative photographs of *Col-0* and *cpl1*-7 germination tests corresponding to Fig. 3b.

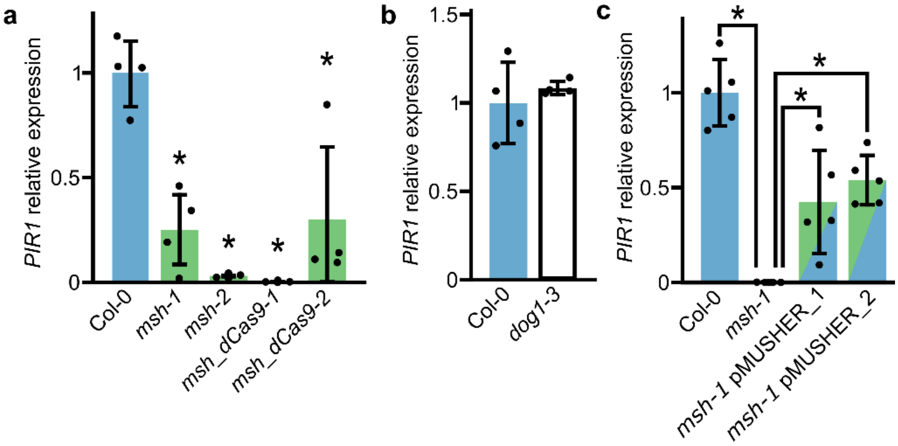


**Supplementary Fig. 5 | *MUSHER* mutants showed decreased *PIR1* expression, which remained unaffected in the *dog1-3* mutant.**

*PIR1* relative expression level in seeds of *msh-1*, *msh-2, msh_dCas9-1*, *msh_dCas9-2* **a** and *dog1-3* **b** mutants subjected to secondary dormancy (2 days), normalised to *UBC21* and relative to WT. *p*-value was calculated in comparison with WT (*$p<0.05$, two-sided *t*-test). Data are presented as the mean value

of four biological replicates +/- SD. **c** Relative expression of *PIR1* in freshly harvested seeds (primary dormancy) of WT, *msh-1* and *msh-1,* and *msh-1 MUSHER-complemented lines (msh-1 pMUSHER_1* and *msh-1 pMUSHER_2)*, normalised to *UBC21* and relative to WT. Data are presented as the mean value of five biological replicates +/- SD (*$p$<0.05, two-sided $t$-test).

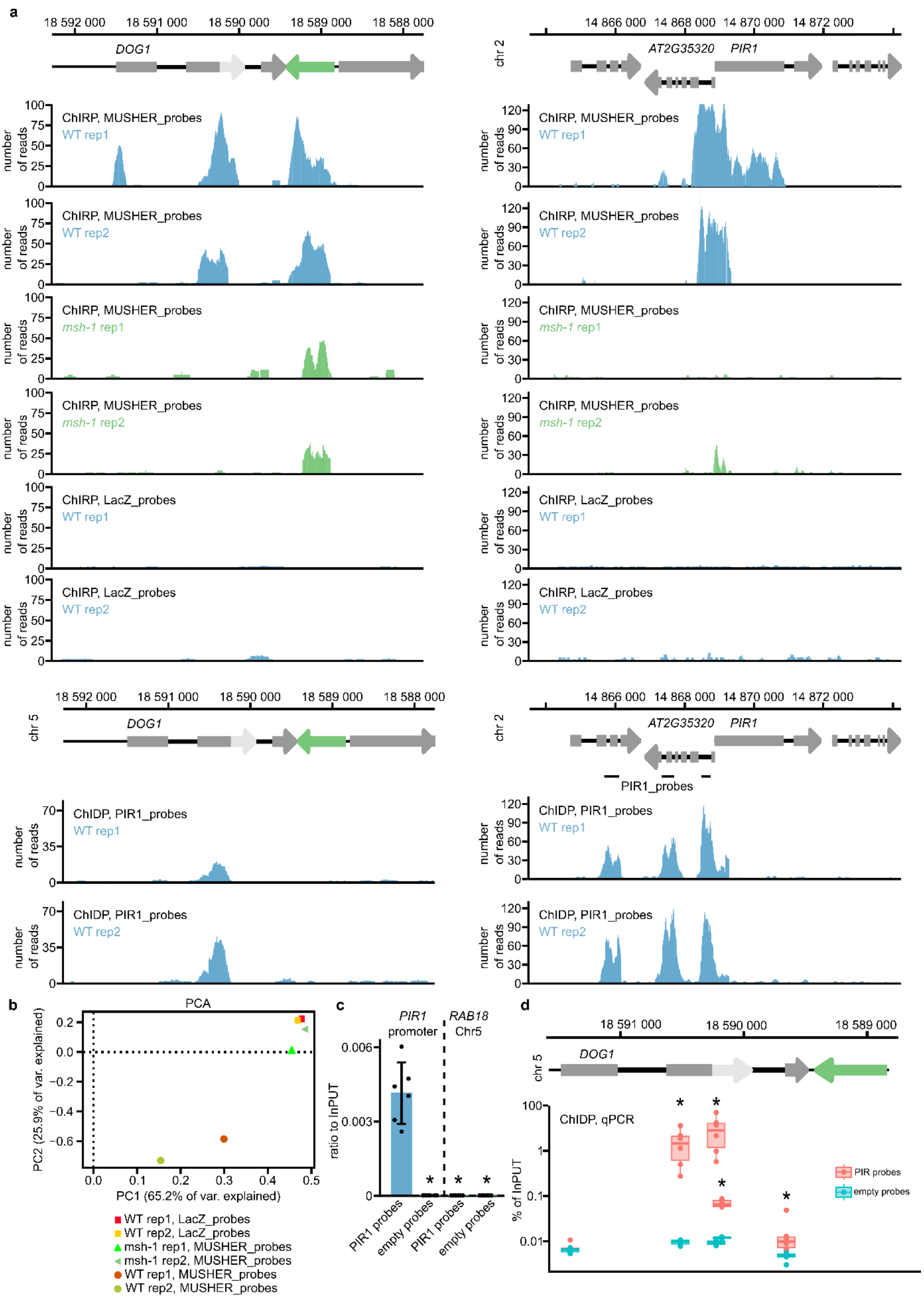
a
18 592 000
18 591 000
18 590 000
18 589 000
18 588 000
DOG1
chr 5
14 866 000
14 868 000
14 870 000
14 872 000
chr 2
AT2G35320
PIR1
number of reads
ChIRP, MUSHER_probes
WT rep1
WT rep2
msh-1 rep1
msh-1 rep2
ChIRP, LacZ_probes
PIR1_probes
ChIDP, PIR1_probes
b
PCA
PC2 (25.9% of var. explained)
PC1 (65.2% of var. explained)
WT rep1, LacZ_probes
WT rep2, LacZ_probes
msh-1 rep1, MUSHER_probes
msh-1 rep2, MUSHER_probes
WT rep1, MUSHER_probes
WT rep2, MUSHER_probes
c
PIR1 promoter
RAB18 Chr5
ratio to InPUT
PIR1 probes
empty probes
d
ChIDP, qPCR
% of InPUT
PIR probes
empty probes

e

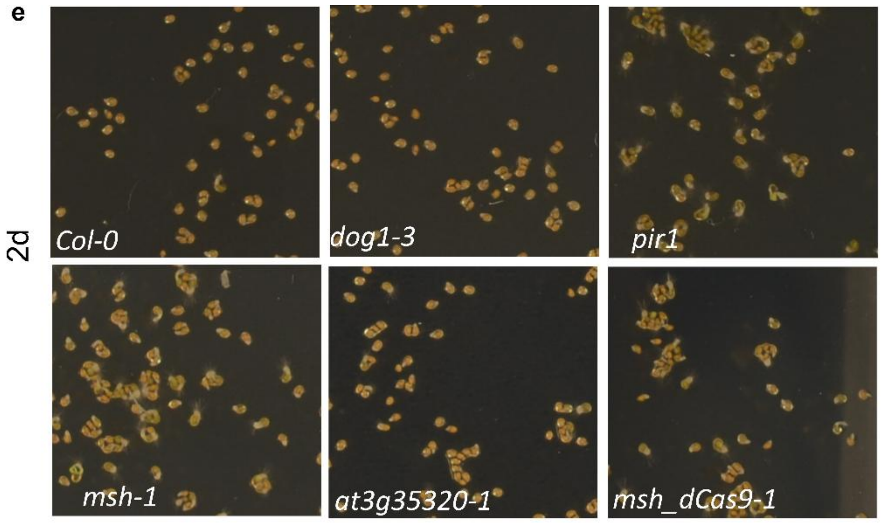
2d
Col-0
dog1-3
pir1
msh-1
at3g35320-1
msh_dCas9-1

f

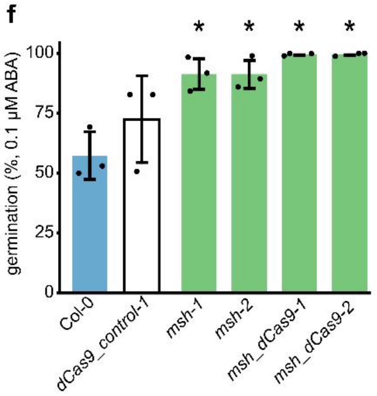
germination (%, 0.1 µM ABA)
100
75
50
25
0
*
Col-0
dCas9_control-1
msh-1
msh-2
msh_dCas9-1
msh_dCas9-2

g

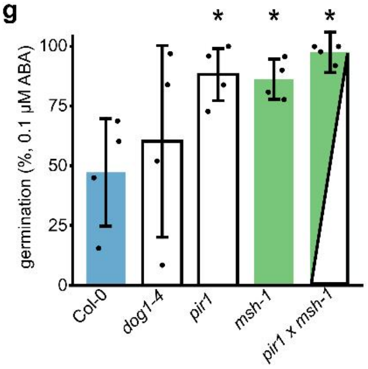
germination (%, 0.1 µM ABA)
100
75
50
25
0
*
Col-0
dog1-4
pir1
msh-1
pir1 x msh-1

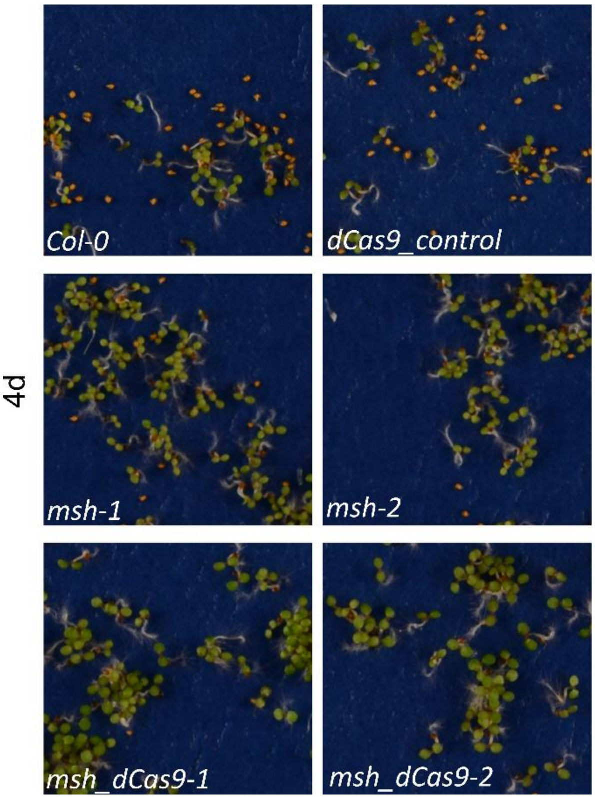
4d
Col-0
dCas9_control
msh-1
msh-2
msh_dCas9-1
msh_dCas9-2

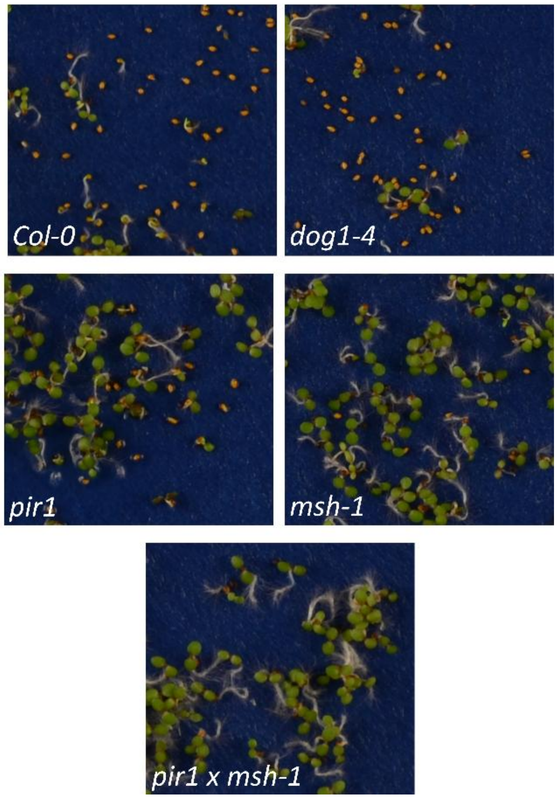
Col-0
dog1-4
pir1
msh-1
pir1 x msh-1

**Supplementary Fig. 6| ChIRP-seq and ChIDP-seq analysis of *MUSHER*, *DOG1*, and *PIR1 loci* in WT and *msh-1* mutants with germination test results.**

**a** Snapshots of ChIRP-seq in WT and *msh-1* mutant using *MUSHER*-specific and *LacZ*-specific probes and ChIDP-seq on *DOG1* and *PIR1 loci*. Schematic diagram showing *DOG1* and *PIR1* locus: exons (grey rectangles), *shDOG1* 3'UTR region (light grey arrow), *lgDOG1* 3'UTR region (grey arrow), and *MUSHER* (green). **b** PCA analysis of ChIRP-seq replicates: MUSHER probes in WT background, lacZ probes in WT, and MUSHER probes in *msh-1*. **c** PIR1 probes bind to the *PIR1* promoter and do not bind to other, randomly chosen *locus* - *RAB18*. Bars are means of 6 replicates, and error bars represent standard deviation. * $p < 0.05$, Mann-Whitney's U-test for comparison with PIR1 probes binding to *PIR1 locus*, a nonparametric test was used since the values are not normally distributed. **d** *PIR1* probes were used to enrich DOG1 from WT, and empty beads mixed with WT chromatin extract (empty probes) were used as a negative control. Box size corresponds to the IQR 25th percentile to 75th percentile. Notches correspond to +/-1.58 IQR/sqrt(n), signifying a 95% confidence interval for the difference in two medians. n=6, * $p<0.05$, Mann-Whitney's U-test for comparing PIR1 to empty probes binding **e** Representative photographs corresponding to the germination tests shown in Fig. 5e. The germination rate of WT, *dCas9-control-1*, *msh-1*, *msh-2, msh_dCas9-1, msh_dCas9-2* **f** and WT, *dog1-4, pir1, msh-1* and double *pir1 x msh-1* **g** mutant seeds on blue paper supplemented with 0,1uM ABA. *P-value<0.05 from two-tailed Welch's t-test. Error bars show standard deviations from three **f** or four **g** biological replicates. Below each chart are representative photographs corresponding to the germination tests above.

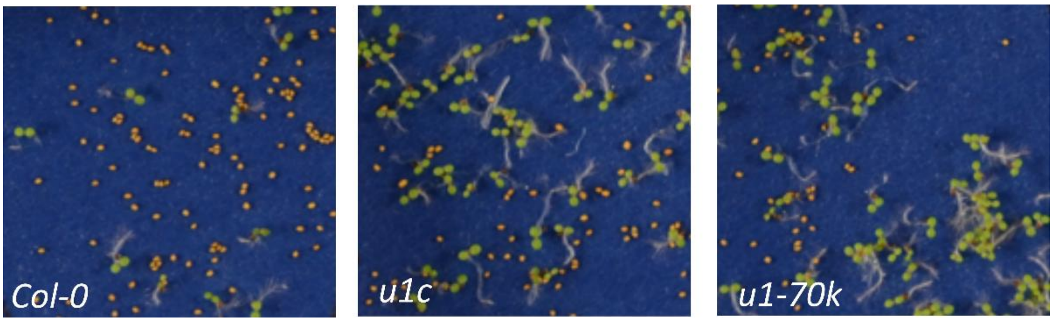


**Supplementary Fig. 7| Lower secondary dormancy of *u1c and u1-70k* mutants.** Representative photographs corresponding to the germination tests shown in Fig. 6e.
