# Supplementary material for: Chromatin retained MUSHER lncRNA integrates ABA and DOG1 signalling pathways to enhance Arabidopsis seeds dormancy
Source: Nat Commun. 2025 Aug 14;16:7545. doi: 10.1038/s41467-025-62991-5 (PMC12354759; doi:10.1038/s41467-025-62991-5)
Supplement: Supplementary file 2 — Description of additional supplementary files [file 41467_2025_62991_MOESM2_ESM.pdf]

Description of Additional Supplementary Files

Supplementary Data 1
MUSHER's 5'RACE and 3'RACE

Supplementary Data 2
Protein coding potential

Supplementary Data 3 Differentially expressed genes in msh-1

Supplementary Data 4
List of MUSHER peaks identified by ChIRP-seq

Supplementary Data 5
List of genomic loci identified with PIR1 utilizing chromatin-DNA precipitation assay (ChIDP-seq)

Supplementary Data 6 Coordinates of predicted U1 binding sites along MUSHER, PUPPIES and COOLAIR lncRNAs

Supplementary Data 7
Primers list
