# Supplementary material for: Chromatin retained MUSHER lncRNA integrates ABA and DOG1 signalling pathways to enhance Arabidopsis seeds dormancy
Source: Nat Commun. 2025 Aug 14;16:7545. doi: 10.1038/s41467-025-62991-5 (PMC12354759; doi:10.1038/s41467-025-62991-5)
Supplement: Supplementary file 4 — Reporting Summary [file 41467_2025_62991_MOESM4_ESM.pdf]

nature portfolio

Corresponding author(s): Szymon Swiezewski

Last updated by author(s): 2025-07-15

Reporting Summary

Nature Portfolio wishes to improve the reproducibility of the work that we publish. This form provides structure for consistency and transparency in reporting. For further information on Nature Portfolio policies, see our Editorial Policies and the Editorial Policy Checklist.

Statistics

For all statistical analyses, confirm that the following items are present in the figure legend, table legend, main text, or Methods section.

n/a	Confirmed	
☐	☒	The exact sample size (*n*) for each experimental group/condition, given as a discrete number and unit of measurement
☐	☒	A statement on whether measurements were taken from distinct samples or whether the same sample was measured repeatedly
☐	☒	The statistical test(s) used AND whether they are one- or two-sided<br>*Only common tests should be described solely by name; describe more complex techniques in the Methods section.*
☒	☐	A description of all covariates tested
☐	☒	A description of any assumptions or corrections, such as tests of normality and adjustment for multiple comparisons
☐	☒	A full description of the statistical parameters including central tendency (e.g. means) or other basic estimates (e.g. regression coefficient) AND variation (e.g. standard deviation) or associated estimates of uncertainty (e.g. confidence intervals)
☐	☒	For null hypothesis testing, the test statistic (e.g. *F*, *t*, *r*) with confidence intervals, effect sizes, degrees of freedom and *P* value noted<br>*Give P values as exact values whenever suitable.*
☒	☐	For Bayesian analysis, information on the choice of priors and Markov chain Monte Carlo settings
☒	☐	For hierarchical and complex designs, identification of the appropriate level for tests and full reporting of outcomes
☒	☐	Estimates of effect sizes (e.g. Cohen's *d*, Pearson's *r*), indicating how they were calculated

Our web collection on statistics for biologists contains articles on many of the points above.

Software and code

Policy information about availability of computer code

Data collection	Sequencing data of MUSHER 5'RACE and 3'RACE were run on Miseq. 3'RNA-seq were run on Illumina NextSeq 500. ChIRP-seq and ChIDP-seq data were run on Illumina NovaSeq 6000. PCR reactions were run with SYBR Green on LightCycler 480 or Biorad.
Data analysis	For ChIRP-seq and ChIDP-seq data analysis, Bowtie2 (v2.5.3)<br>For 5'RACE and 3'RACE-seq data analysis, STAR (v2.7.8a) was used to align the reads to the reference genome, followed by UMI-based PCR duplicates removal using UMI-tools (v1.1.0). The position of the first 5′ nucleotide or the last 3′ nucleotide was extracted using bedtools (v2.30.0).<br>For 3'RNAseq alignment and read demµltiplexing were performed using STAR (v2.7.8a) and BRBseqTools (v 1.6), respectively. Differentially expressed genes were identified using DESeq2 with a cutoff of FDR < 0.05 and FC > 1.<br>For smFISH analysis cells were manually segmented using Napari and the PartSeq plugin, which allows foci identification and classification based on subcellular location. Dots number and intensity were analysed using R scripts described earlier in (Montez, M. et al. Promoter-pervasive transcription causes RNA polymerase II pausing to boost DOG1 expression in response to salt. EMBO J 42, e112443 (2023)<br>For RTqPCR analysis and germination test calculation MS Office Excel was used.<br>Figures were created in Inkscape v1.3.2

For manuscripts utilizing custom algorithms or software that are central to the research but not yet described in published literature, software must be made available to editors and reviewers. We strongly encourage code deposition in a community repository (e.g. GitHub). See the Nature Portfolio guidelines for submitting code & software for further information.

Data

Policy information about availability of data

All manuscripts must include a data availability statement. This statement should provide the following information, where applicable:

- Accession codes, unique identifiers, or web links for publicly available datasets
- A description of any restrictions on data availability
- For clinical datasets or third party data, please ensure that the statement adheres to our policy

Sequencing data has been deposited at the Gene Expression Omnibus (GEO) under the accession code GSE274757. A full list of PCR primers is available in Supplementary Table 1. The raw data of RT-qPCR, ChIP-qPCR, RIP-qPCR are available in Source Data. The TAIR accession numbers for the genes analysed in this study are DOG1 (AT5G45830), PIR1 (AT2G35330), CPL1 (AT4G21670), U1-70K (AT3G50670). Standard reference gene UBC (AT5G25760), Actin 2/7 (AT5G09810) were used for normalization of gene expression and ChIP data respectively.

Research involving human participants, their data, or biological material

Policy information about studies with human participants or human data. See also policy information about sex, gender (identity/presentation), and sexual orientation and race, ethnicity and racism.

Reporting on sex and gender	*Use the terms sex (biological attribute) and gender (shaped by social and cultural circumstances) carefully in order to avoid confusing both terms. Indicate if findings apply to only one sex or gender; describe whether sex and gender were considered in study design; whether sex and/or gender was determined based on self-reporting or assigned and methods used. Provide in the source data disaggregated sex and gender data, where this information has been collected, and if consent has been obtained for sharing of individual-level data; provide overall numbers in this Reporting Summary. Please state if this information has not been collected. Report sex- and gender-based analyses where performed, justify reasons for lack of sex- and gender-based analysis.*
Reporting on race, ethnicity, or other socially relevant groupings	*Please specify the socially constructed or socially relevant categorization variable(s) used in your manuscript and explain why they were used. Please note that such variables should not be used as proxies for other socially constructed/relevant variables (for example, race or ethnicity should not be used as a proxy for socioeconomic status). Provide clear definitions of the relevant terms used, how they were provided (by the participants/respondents, the researchers, or third parties), and the method(s) used to classify people into the different categories (e.g. self-report, census or administrative data, social media data, etc.) Please provide details about how you controlled for confounding variables in your analyses.*
Population characteristics	*Describe the covariate-relevant population characteristics of the human research participants (e.g. age, genotypic information, past and current diagnosis and treatment categories). If you filled out the behavioural & social sciences study design questions and have nothing to add here, write "See above."*
Recruitment	*Describe how participants were recruited. Outline any potential self-selection bias or other biases that may be present and how these are likely to impact results.*
Ethics oversight	*Identify the organization(s) that approved the study protocol.*

Note that full information on the approval of the study protocol must also be provided in the manuscript.

Field-specific reporting

Please select the one below that is the best fit for your research. If you are not sure, read the appropriate sections before making your selection.

☒ Life sciences ☐ Behavioural & social sciences ☐ Ecological, evolutionary & environmental sciences

For a reference copy of the document with all sections, see nature.com/documents/nr-reporting-summary-flat.pdf

Life sciences study design

All studies must disclose on these points even when the disclosure is negative.

Sample size	No statistical approach was used to predetermine sample size. Sample size were determined based on previous publications on similar previous experiments. The determined sample size was adeqaute as the differences between experimental groups was significant and reproducible. RT-qPCR (e.g. Fedak, H. et al. Control of seed dormancy in Arabidopsis by a cis-acting noncoding antisense transcript. Proc. Natl. Acad. Sci. U.S.A. 113, E7846–E7855 (2016). ChIP-qPCR (Kowalczyk, J. et al. Alternative Polyadenylation of the Sense Transcript Controls Antisense Transcription of DELAY OF GERMINATION 1 in Arabidopsis. Molecular Plant 10, 1349–1352 (2017). Fractionation (Montez, M. et al. Promoter-pervasive transcription causes RNA polymerase II pausing to boost DOG1 expression in response to salt. EMBO J 42, e112443 (2023)
Data exclusions	No data were excluded.
Replication	The main conclusions were validated through multiple assays, including expression analysis, ChIRP, ChIP, RIP, and ChIDP. Additionally, we utilized different mutants to support our findings. Each experiment was conducted with at least four independent biological replicates.

Randomization	Plants of different genotypes were grown side by side to minimize unexpected environmental variations during growth and experimentation. Seeds/seedlings at the same developmental stage were collected and assessed randomly for each genotype/treatment. For ChIRP-qPCR, multiple seeds were randomly collected from different plants for each replicate. For RNA expression, multiple, randomly selected individual plants were collected from a plate for each replicate.
Blinding	Blinding was not required for the molecular biology techniques, as all samples were handled simultaneously and under identical conditions, eliminating the possibility of bias.

Reporting for specific materials, systems and methods

We require information from authors about some types of materials, experimental systems and methods used in many studies. Here, indicate whether each material, system or method listed is relevant to your study. If you are not sure if a list item applies to your research, read the appropriate section before selecting a response.

Materials & experimental systems

n/a	Involved in the study
☐	☒ Antibodies
☒	☐ Eukaryotic cell lines
☒	☐ Palaeontology and archaeology
☒	☐ Animals and other organisms
☒	☐ Clinical data
☒	☐ Dual use research of concern
☐	☒ Plants

Methods

n/a	Involved in the study
☒	☐ ChIP-seq
☒	☐ Flow cytometry
☒	☐ MRI-based neuroimaging

Antibodies

Antibodies used	anti-RPB1 | RNA polymerase II subunit B1 (Agrisera, AS11 1804), GFP-trap (Chromotek, gta-10); anti-H3 (Abcam, ab1791); anti-H3K27me3 (Millipore, 07-449), anti-H3K4me3 (Millipore, 07-473) anti CPSF73 (PhytoAB, PHY1043A)
Validation	Antibodies used for CoIP, ChIP, WB. Validation was as published and done by commercial providers (CST, Abcam, Millipore, Chromotek, Sigma, Agrisera).

Dual use research of concern

Policy information about dual use research of concern

Hazards

Could the accidental, deliberate or reckless misuse of agents or technologies generated in the work, or the application of information presented in the manuscript, pose a threat to:

No	Yes
☒	☐ Public health
☒	☐ National security
☒	☐ Crops and/or livestock
☒	☐ Ecosystems
☒	☐ Any other significant area

Experiments of concern

Does the work involve any of these experiments of concern:

No	Yes	
☒	☐	Demonstrate how to render a vaccine ineffective
☒	☐	Confer resistance to therapeutically useful antibiotics or antiviral agents
☒	☐	Enhance the virulence of a pathogen or render a nonpathogen virulent
☒	☐	Increase transmissibility of a pathogen
☒	☐	Alter the host range of a pathogen
☒	☐	Enable evasion of diagnostic/detection modalities
☒	☐	Enable the weaponization of a biological agent or toxin
☒	☐	Any other potentially harmful combination of experiments and agents

Plants

Seed stocks	*Report on the source of all seed stocks or other plant material used. If applicable, state the seed stock centre and catalogue number. If plant specimens were collected from the field, describe the collection location, date and sampling procedures.*
Novel plant genotypes	*Describe the methods by which all novel plant genotypes were produced. This includes those generated by transgenic approaches, gene editing, chemical/radiation-based mutagenesis and hybridization. For transgenic lines, describe the transformation method, the number of independent lines analyzed and the generation upon which experiments were performed. For gene-edited lines, describe the editor used, the endogenous sequence targeted for editing, the targeting guide RNA sequence (if applicable) and how the editor was applied.*
Authentication	*Describe any authentication procedures for each seed stock used or novel genotype generated. Describe any experiments used to assess the effect of a mutation and, where applicable, how potential secondary effects (e.g. second site T-DNA insertions, mosiacism, off-target gene editing) were examined.*
